# RNA Sequencing and Targeted Knockdown Reveal miR-142a-5p as a Driver of Retinal Degeneration in rd1 Mice

**DOI:** 10.3390/biology15020134

**Published:** 2026-01-13

**Authors:** Na Yang, Meng Zhao, Nan Guo, Mei Yang, Yanli Ji, Xin Wang, Lirong Zhang, Ji Xu, Guang-Hua Peng

**Affiliations:** 1 Laboratory of Visual Cell Differentiation and Regulation, Basic Medical College, Zhengzhou University, Zhengzhou 450001, China; 2 Department of Pathophysiology, Basic Medical College, Zhengzhou University, Zhengzhou 450001, China; 3 Department of Pharmacology, Basic Medical College, Zhengzhou University, Zhengzhou 450001, China

**Keywords:** retinitis pigmentosa, retinal degeneration, microRNA, rd1

## Abstract

Retinitis pigmentosa (RP), an inherited retinal disorder, leads to progressive photoreceptor degeneration and irreversible blindness, with limited treatment options available. Emerging evidence implicates microRNAs (miRNAs) in the pathogenesis of retinal disease, yet understanding of their specific roles in RP remains incomplete. In this study, we employed high-throughput RNA sequencing to profile miRNA expression in a rd1 RP mouse model at postnatal day 14. Our analysis revealed 40 upregulated and 27 downregulated miRNAs in rd1 retinas compared to controls. Notably, miR-142a-5p, miR-223-3p, and miR-653-5p were significantly elevated, while miR-25-3p was downregulated. Given miR-142a-5p’s established roles in apoptosis and inflammation, we investigated its contribution to retinal degeneration. Knockdown of miR-142a-5p in rd1 mice improved retinal function and preserved outer nuclear layer thickness, suggesting a protective effect against photoreceptor loss. These findings highlight miR-142a-5p as a key regulator of RP progression and a promising therapeutic target for mitigating vision loss in retinal degenerative diseases.

## Simple Summary

Inherited eye diseases can cause the light-sensing cells to break down, leading to permanent blindness; there are very few treatments available. Our research focused on understanding the role of microRNAs, a kind of small RNA, which act as switches that can turn the expression of certain genes off inside cells. Using a mouse model of an inherited eye disease called retinitis pigmentosa, we discovered that the amounts of several of these microRNAs can either increase or decrease. One in particular, named miR-142, significantly increased in amount during disease progression. When we used a special tool to lower the amount of miR-142 in the eyes of these mice, we saw that the disease progressed more slowly; the mice retained more of their light-sensing cells and had better vision. This implies that miR-142 plays a key role in driving vision loss. Our study suggests that developing a drug to block miR-142 could be a promising new strategy for treating this type of blindness.

## 1. Introduction

Retinitis pigmentosa (RP) is caused by genetic mutations, with over 90 genes identified as contributors to the disease [1]. Clinically, RP initially presents as night blindness, followed by the loss of central vision and, ultimately, complete blindness [2]. The global incidence of RP is approximately 1 in 4000 individuals [3]. Although gene therapy has made significant breakthroughs in RP treatment, many challenges remain in developing effective therapies [4].

In the early stages of RP, rod photoreceptor degeneration occurs first, followed by cone cell death. Several theories have been proposed to explain the mechanisms underlying cone cell degeneration secondary to rod cell loss. One theory suggests that rod cell death leads to increased oxygen levels in the outer retina. This elevated oxygen content generates a large amount of oxygen free radicals, resulting in oxidative damage and eventual cone cell death [5]. Another theory posits that rods produce rod-derived cone viability factor (RdCVF), which mediates glucose uptake and stimulates aerobic glycolysis to promote cone survival [6]. Following rod cell degeneration and death, the secretion of RdCVF decreases, accelerating the death of cone cells [7].

MicroRNAs (miRNAs) are endogenous non-coding small RNA molecules, approximately 21–25 nucleotides in length, that are encoded by the genomes of higher eukaryotes. Their expression is temporary and tissue-specific, and they play a crucial role in regulating post-transcriptional gene expression [8]. miRNAs are essential for maintaining retinal cell homeostasis, function, and survival. For instance, miR-204-5p has been shown to inhibit autophagy in diabetic retinopathy by downregulating the expression of microtubule-associated protein 1 light chain 3B-II (LC3B-II) [9]. In models of photooxidative retinal damage, miR-124 reduces retinal inflammation and improves retinal function by modulating chemokine ligand 2 (CCL2) [10]. Additionally, it has been found that knocking down miR-6937-5p can slow the deterioration of visual function in the retinas of rd10 mice [11].

miRNA mimics and inhibitors are promising therapeutic candidates for RP, making comprehensive miRNA expression profiling a critical first step in their development. While previous studies have characterized RP-associated miRNA signatures using microarray platforms followed by qPCR validation, the advent of high-throughput sequencing now enables the detection of subtle miRNA expression changes with substantially greater resolution [12,13]. Although miRNA-seq has been employed to profile miRNAs in naïve retina and other retinal disorders, it has not yet been applied to RP [14,15,16]. In this study, we performed systematic miRNA-seq analysis of RP model retinas to identify dysregulated miRNAs, validated candidates using qPCR, and pinpointed key drivers of disease progression.

To identify potential new targets for RP therapy, we focused on rd1 mice, a classic RP animal model. The rd1 mouse model is characterized by mutations in the *Pde6b* gene, located on chromosome 5, which follows an autosomal recessive inheritance pattern. Mutations in *Pde6b* result in insufficient phosphodiesterase activity in rod cells, leading to the accumulation of the second messenger cyclic guanosine monophosphate (cGMP). This accumulation disrupts the closure of cGMP-gated channels, ultimately causing rod cell death [17].

In this study, we analyzed miRNA expression patterns during the early and peak stages of retinal degeneration in rd1 mice. We identified several miRNAs with potential therapeutic significance for RP, including miR-142a-5p and miR-223-3p, which have been implicated in other retinal diseases, as well as miR-653-5p and miR-25-3p. Notably, we found that inhibiting miR-142a-5p expression delayed the degeneration of retinal photoreceptor cells and improved retinal function in rd1 mice. These findings suggest that miR-142a-5p could serve as a promising new target for the treatment of RP.

## 2. Materials and Methods

### 2.1. Ethics Statement and Animal Handling

C57BL/6J (wild-type control) and rd1 (Pde6brd1 mutant, RRID: IMSR_JAX:004766) mice were maintained under specific pathogen-free conditions at 22–24 °C with 40–50% humidity and a 12 h light/dark cycle. C57BL/6J mice were obtained from Beijing Vital River Laboratory Animal Technology (Beijing, China), while rd1 mice were acquired from The Jackson Laboratory (Bar Harbor, ME, USA). All procedures were performed in accordance with China’s Regulations for the Administration of Affairs Concerning Experimental Animals and approved by Zhengzhou University’s Animal Ethics Committee (approval #ZZUIRB2022-075; 10 July 2022).

### 2.2. Histological Assessment

Mice were euthanized using cervical dislocation, and their eyeballs were enucleated and fixed in 4% paraformaldehyde (LABGIC, Anhui, China) for 48 h at 4 °C. Following fixation, the anterior segment and lens were removed to isolate the retinal eyecup. Tissues were dehydrated through a graded ethanol series (70%, 80%, 90%, 95%, and 100%), cleared in xylene, and embedded in paraffin wax (SAKURA/TEC, Tokyo, Japan). Serial sections (4 μm thickness) were cut along the optic nerve axis using a rotary microtome (YAMATO RX-860, Tokyo, Japan), with three representative sections retained per eye. The sections were baked at 65 °C for 1 h, dewaxed in xylene, and rehydrated through a descending alcohol series (100%, 95%, and 70%) to distilled water. Tissue sections were stained with hematoxylin (20 s) and eosin (10 s), dehydrated through an ascending alcohol series, cleared in xylene, and mounted with neutral balsam. Bright-field images were captured at 40× magnification (Olympus BX53, Tokyo, Japan) at 200–300 μm from the optic nerve head. Outer nuclear layer (ONL) thickness was measured using ImageJ software (version 1.53t, NIH, Bethesda, MD, USA) with three independent measurements per section for statistical analysis.

### 2.3. TUNEL Staining

To assess photoreceptor apoptosis, retinal paraffin sections were processed using a TUNEL staining kit (Beyotime, Shanghai, China) according to the manufacturer’s protocol. The sections were first baked at 60 °C for 1 h, dewaxed in xylene, and then rehydrated through a graded ethanol series to distilled water. Tissue areas were delineated using an immunohistochemical barrier pen, followed by proteinase K digestion (20 μL of 20 μg/mL) for 30 min at room temperature. After three 5 min PBS washes, sections were incubated with TUNEL reaction mixture (Beyotime, Shanghai, China) (20 μL/section, prepared at a 1:9 ratio of TdT enzyme to fluorescent labeling solution) for 60 min at 37 °C in a humidified dark chamber. Following three additional 5 min PBS washes, nuclei were counterstained with DAPI (1 μg/mL, 5 min), and slides were mounted with anti-fade mounting medium. Fluorescence images were acquired using a 40× objective on a fluorescence microscope (Olympus, Tokyo, Japan) (excitation/emission: 488/530 nm for TUNEL, 358/461 nm for DAPI).

### 2.4. Electroretinography (ERG) Recording

Following overnight dark adaptation (>12 h), the mice were anesthetized using isoflurane (RWD; 3% induction, 1.5% maintenance) and positioned on a temperature-regulated platform. Their pupils were dilated with compound tropicamide eye drops (Santen OY, Osaka, Japan), and corneal hydration was maintained with sodium hyaluronate (Santen OY) (Santen OY, Osaka, Japan). Electrodes were placed as follows: a gold loop corneal electrode as the active electrode, subdermal needle electrodes in both cheeks as references, and a ground electrode at the tail base. Full-field flash ERG was recorded using a Roland Consult system (Roland Consult, Heidelberger, Germany) with standardized flash intensities (0.01 cd·s/m^2^ for scotopic and 3.0 cd·s/m^2^ for mixed responses) and 10 min inter-stimulus intervals. The signals were amplified (×1000), bandpass-filtered (0.3–500 Hz), and sampled at 2 kHz. A-wave and b-wave amplitudes were analyzed from at least three mice per group using manufacturer-supplied software (RETIanalysis, version 5.2).

### 2.5. Sample Collection and Processing

Retinal tissues were collected from rd1 and wild-type C57BL/6J mice at postnatal days 9 (P9) and 14 (P14). Following euthanasia by cervical dislocation on ice, their eyes were immediately enucleated and dissected in RNAlater solution under RNAse-free conditions. Each biological replicate consisted of pooled retinal tissues from both eyes of a single animal (*n* = 3 mice per group), flash-frozen in liquid nitrogen for 15 min, and stored at −80 °C until processing. Samples were shipped on dry ice to Wuhan Kangce Technology Co., Ltd. for miRNA sequencing (Illumina HiSeq X-10, PE150 mode, Wuhan, China). The experimental groups were designated as E1-E3 (P14 rd1), C1-C3 (P14 C57BL/6J), E4-E6 (P9 rd1), and C4-C6 (P9 C57BL/6J), with each sample processed independently through library preparation and sequencing.

### 2.6. Quantitative PCR Validation

Differentially expressed miRNAs identified by sequencing were validated using stem-loop RT-qPCR. Retinal tissues from P9 and P14 rd1 mice were homogenized in TRIzol (Beyotime, R0016, Shanghai, China) on ice, and total RNA was extracted following the manufacturer’s protocol. RNA concentration and purity were assessed by spectrophotometry (A260/A280 ratio > 1.9).

Reverse transcription of miRNA was performed using miRNA-specific stem-loop primers. Quantitative PCR with PowerUp SYBR Green Master Mix (Thermo Fisher, A25742, Waltham, MA, USA) was performed on a QuantStudio 6 Flex system. Cycling conditions: 95 °C for 2 min, followed by 40 cycles of 95 °C for 15 sec, 55–60 °C for 15 sec, and 72 °C for 1 min. All reactions were run in technical triplicate with U6 snRNA as the endogenous control. Primer sequences for reverse transcription and forward primer for PCR are provided in Supplementary Table S1. Common reverse primer: GTGCAGGGTCCGAGGT. Data were analyzed using the $2^{-\Delta \Delta Ct}$ method. Quantification of miRNA by the TaqMan method was performed using Hieff Unicon^®^ Pure Pro U+ qPCR Mix (Yeasen #16713ES60, Shanghai, China). Cycling conditions: 95 °C for 10 min, followed by 40 cycles of 95 °C for 15 s, and 60 °C for 1 min. TaqMan probe sequence for miRNA-1422-5p: 5′ FAM-ACTACTGTCGTATCCAGT- MGBNFQ 3′, TaqMan probe sequence for U6: 5CGATACAGAGAAGATTAGCATGGC. Data were analyzed using the $2^{-\Delta \Delta Ct}$ method.

Reverse transcription of mRNA was performed using RevertAid First Strand cDNA Synthesis Kit (Thermo Scientific™, K16225, Waltham, MA, USA). Quantitative PCR with PowerUp SYBR Green Master Mix (Thermo Fisher, A25742, Waltham, MA, USA) was performed on a QuantStudio 6 Flex system. Cycling conditions: 95 °C for 2 min, followed by 40 cycles of 95 °C for 15 sec, 55–60 °C for 15 sec, and 72 °C for 1 min. Primer sequences for PCR are provided in Supplementary Table S2. All reactions were run in technical triplicate with GAPDH as the endogenous control. Data were analyzed using the $2^{-\Delta \Delta Ct}$ method.

### 2.7. miRNA Expression Profiling and Bioinformatics Analysis

Differentially expressed miRNAs were identified using thresholds of |log2(fold change)| ≥ 1.5 and adjusted *p*-value < 0.05. The candidate miRNAs were prioritized based on their established roles in retinal degeneration through a review of the literature. Experimentally validated miRNAs were subjected to target prediction using RNAhybrid (v2.1.2) and miRanda (v3.3a) algorithms with default parameters (minimum free energy ≤ −20 kcal/mol for RNAhybrid; score ≥ 140 and energy ≤ −10 kcal/mol for miRanda). Predicted targets were further filtered for conservation across mammals (PhastCons score ≥ 0.5). Interaction networks were visualized using Cytoscape (v3.8.2), with nodes representing miRNAs/mRNAs and edges indicating predicted interactions supported by both algorithms.

### 2.8. Construction of the scAAV Virus

Fragments carrying microRNA-142a-5p blocking sequences were cloned into the H13782 pscAAV-U6-shRNA-CMV-EGFP-tWPA adeno-associated viral vector to form the recombinant expression vector pscAAV-U6-Decoy (mmu-microRNA-142a-5p)-CMV-EGFP-tWPA, and pcscAAV-tCMV-EGFP-tWPA was used as the control virus. The serotype was AAV2/5. The preparation of adeno-associated virus vectors was completed by OBIO Technology (Shanghai, China).

### 2.9. Subretinal Injection

A total of twelve P9 rd1 mice (mixed-sex cohorts) were divided equally into experimental and control groups, receiving either miR-142a-5p-interfering scAAV or control scAAV (1 μL of 1 × 10^12^ vg/mL solution) via bilateral subretinal injections. Under isoflurane anesthesia (RWD Life Science Co., Ltd., Shenzhen, China) (3% induction, 1.5% maintenance), their pupils were dilated with compound tropicamide eye drops (Santen OY) followed by topical application of proparacaine hydrochloride (0.5%) (Santen, Osaka, Japan) for local anesthesia. Using a 33 G beveled needle to create scleral access at the corneal limbus, viral suspensions were delivered through a Hamilton microsyringe (Hamilton Laboratory Equipment, Shanghai, China) at two injection sites per eye. Postoperative care included topical antibiotic application and daily monitoring for ocular complications.

### 2.10. Preparation of Frozen Sections and DAPI Staining

Eyeballs were punctured at the corneal limbus under microscopic guidance and fixed in 4% paraformaldehyde for 2 h at 4 °C. Following anterior segment removal and lens extraction, the eyecups were cryoprotected in 30% sucrose overnight at 4 °C before embedding in optimal cutting temperature compound (SAKURA #4583, Tokyo, Japan). Frozen sections (10–12 μm thickness) were air-dried at room temperature for 10–15 min, then washed 3× with PBS. Retinal sections were encircled with a hydrophobic barrier pen and permeabilized with 0.3% Triton X-100 (Solarbio #T8200, Beijing, China) in PBS (20 μL/section) for 15 min at room temperature. After removing excess Triton solution, the nuclei were stained with DAPI (10 μL/section, 5 min, RT), followed by 3× PBS washes. Sections were mounted with anti-fade medium and imaged using a fluorescence microscope (40× objective).

### 2.11. Statistical Analysis

The statistical analysis was processed with GraphPad Prism 7.0 software. The experimental data were presented as mean ± standard deviation (Mean ± SD). The differences between two or more sets of data were compared using statistical methods such as Student’s *t*-test or two-way ANOVA.

## 3. Results

### 3.1. The Thickness of the Retinal Outer Nuclear Layer in rd1 Mice Gradually Decreased over Time

**Figure 1:**
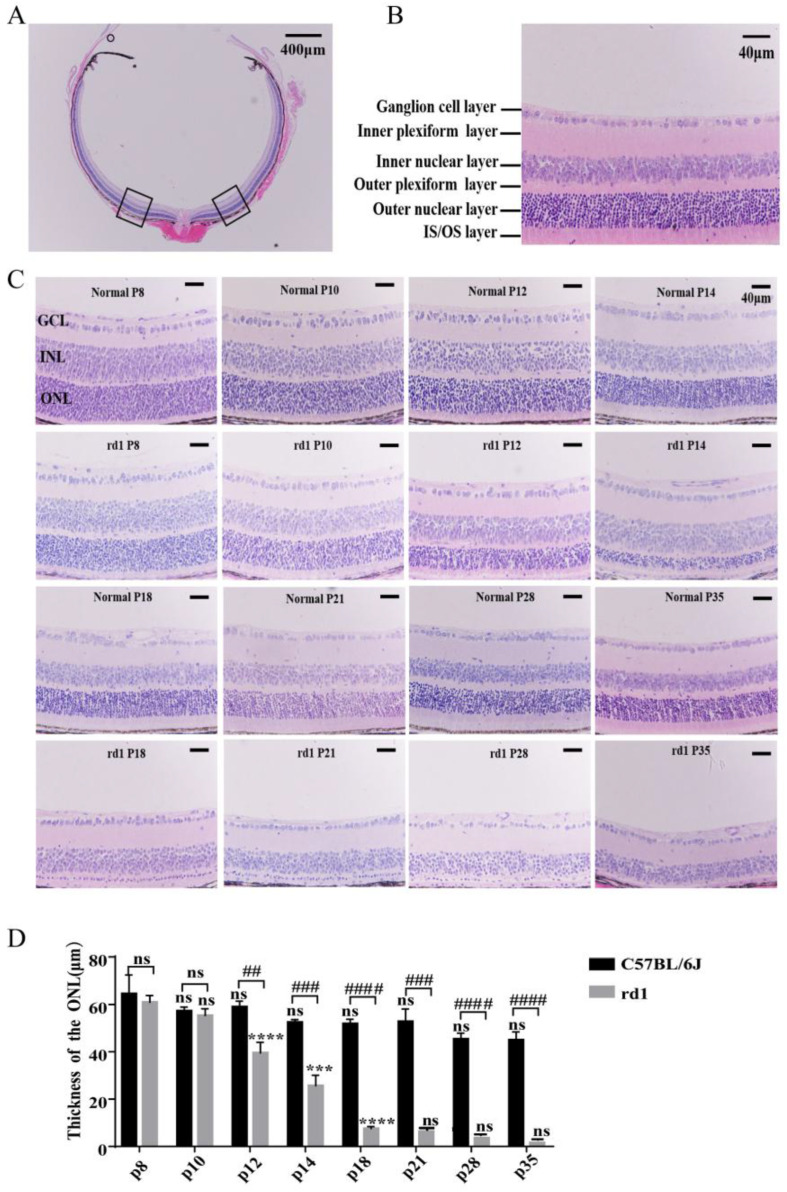
Progressive retinal degeneration in rd1 mice. (**A**) Low-magnification view of retinal morphology (10×; scale bar = 400 μm). Boxes show the positions where pictures as in panel B were taken. (**B**) Laminar organization of wild-type retina showing distinct layers (40×; scale bar = 40 μm). (**C**) Comparative retinal sections from rd1 and age-matched control mice at indicated postnatal days (*p*) (40×; scale bar = 40 μm). (**D**) Quantification of ONL thickness showing progressive thinning in rd1 mice. Data represent mean ± SD (*n* = 3 mice/group). *** *p* < 0.001, **** *p* < 0.0001 by two-way ANOVA (vs. previous time point); ## *p* < 0.01, ### *p* < 0.001, #### *p* < 0.0001 by unpaired *t*-test (vs. age-matched controls); ns: not significant.

To assess morphological changes, we performed hematoxylin and eosin (H&E) staining on retinal tissues from rd1 and normal mice at postnatal days 8, 10, 12, 14, 18, 21, 28, and 35 (Figure 1A,B). Two-way ANOVA revealed that the thickness of the ONL in rd1 mice progressively decreased over time, almost completely disappearing by day 35 (Figure 1C,D). Unpaired *t*-test results demonstrated that the ONLs of rd1 mice were significantly thinner compared to age-matched normal mice starting from postnatal day 12 (Figure 1C,D; *p* < 0.01). These findings indicate that the photoreceptor cells in the ONLs of rd1 mice were severely damaged shortly after birth, with degeneration progressively worsening until the ONL was almost entirely lost.

### 3.2. Retinal Function in rd1 Mice Is Severely Impaired over Time

**Figure 2:**
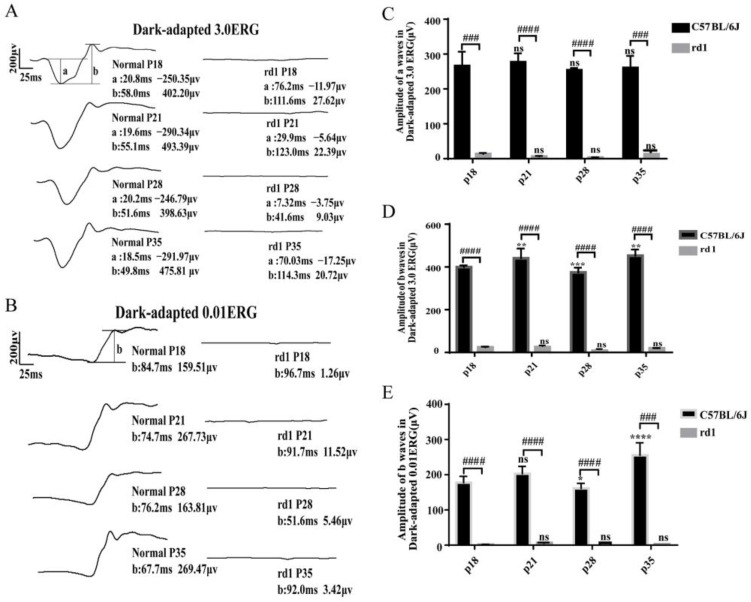
Functional assessment of retinal degeneration in rd1 mice using ERG. (**A**,**B**) Representative ERG waveforms under (**A**) dark-adapted 3.0 cd·s/m^2^ (mixed rod–cone response) and (**B**) dark-adapted 0.01 cd·s/m^2^ (rod-dominated response) stimuli at indicated postnatal days. (**C**,**D**) Quantification of (**C**) a-wave (photoreceptor activity) and (**D**) b-wave (bipolar cell activity) amplitudes from 3.0 cd·s/m^2^ stimuli. (**E**) B-wave amplitude analysis from 0.01 cd·s/m^2^ stimuli. Data represent mean ± SD (*n* = 3 mice/group). * *p* < 0.05, ** *p* < 0.01, *** *p* < 0.001, **** *p* < 0.0001 by two-way ANOVA (vs. previous time point); ### *p* < 0.001, #### *p* < 0.0001 by unpaired *t*-test (vs. age-matched controls); ns: not significant.

The full-field electroretinogram (ffERG) amplitude serves as a reliable indicator of retinal function and is crucial for monitoring retinal diseases [18]. To evaluate changes in retinal function, we performed ffERG tests on rd1 and normal mice at postnatal days 18, 21, 28, and 35. Unpaired *t*-test results revealed that retinal function in rd1 mice was significantly impaired by postnatal day 18 compared to age-matched normal mice (Figure 2). These findings are consistent with the morphological changes observed in H&E staining, further demonstrating the progressive functional decline in rd1 mice.

### 3.3. Apoptotic Changes in the Outer Nuclear Layers of rd1 Mouse Eyes

**Figure 3:**
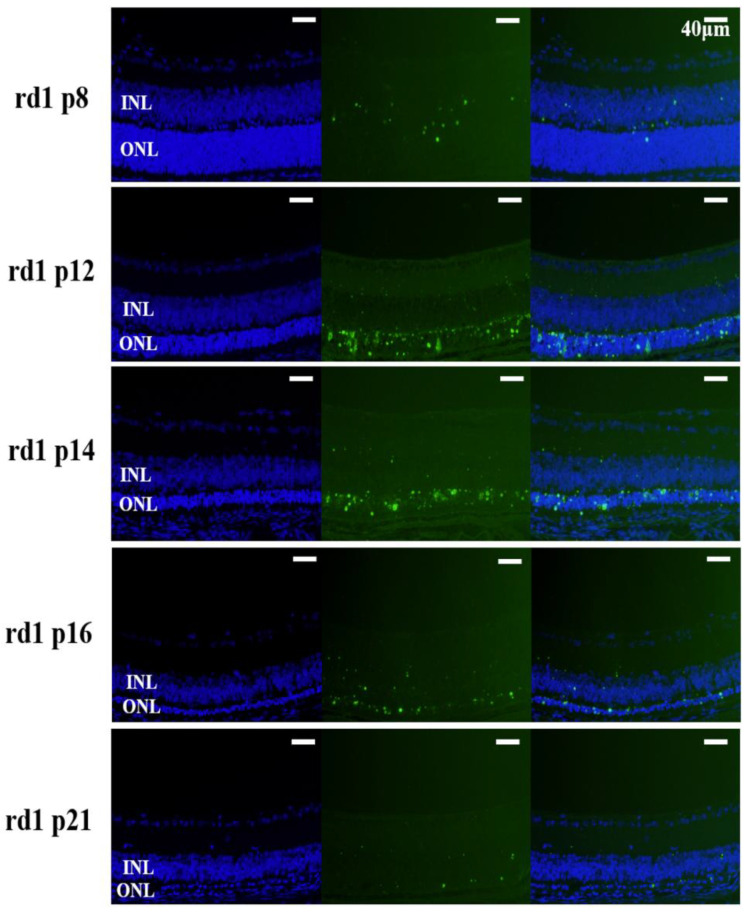
Temporal progression of photoreceptor apoptosis in rd1 mice. Representative TUNEL/DAPI-stained retinal sections at indicated postnatal days (40× magnification; scale bar = 40 μm) of rd1 mice. Blue: DAPI nuclear counterstain (all nuclei). Green: TUNEL-positive cells (apoptotic nuclei). Note peak apoptosis at P14 with residual apoptotic cells persisting at P21 despite ONL thinning.

To assess apoptosis in retinal photoreceptor cells, we performed TUNEL staining on retinal paraffin sections from rd1 mice at postnatal days 8, 12, 14, 16, and 21. We observed that photoreceptor cells in the ONLs of rd1 mice began to undergo apoptosis by postnatal day 8. The number of apoptotic cells peaked on postnatal day 14. By postnatal day 21, only a single layer of photoreceptor cells remained, and apoptotic activity was still evident (Figure 3 and Figure S1). In contrast, there is no detectable apoptotic cell in the sections from WT mice (Figure S2).

### 3.4. Expression Changes in miRNA Profiles in rd1 Mouse Retina

**Figure 4:**
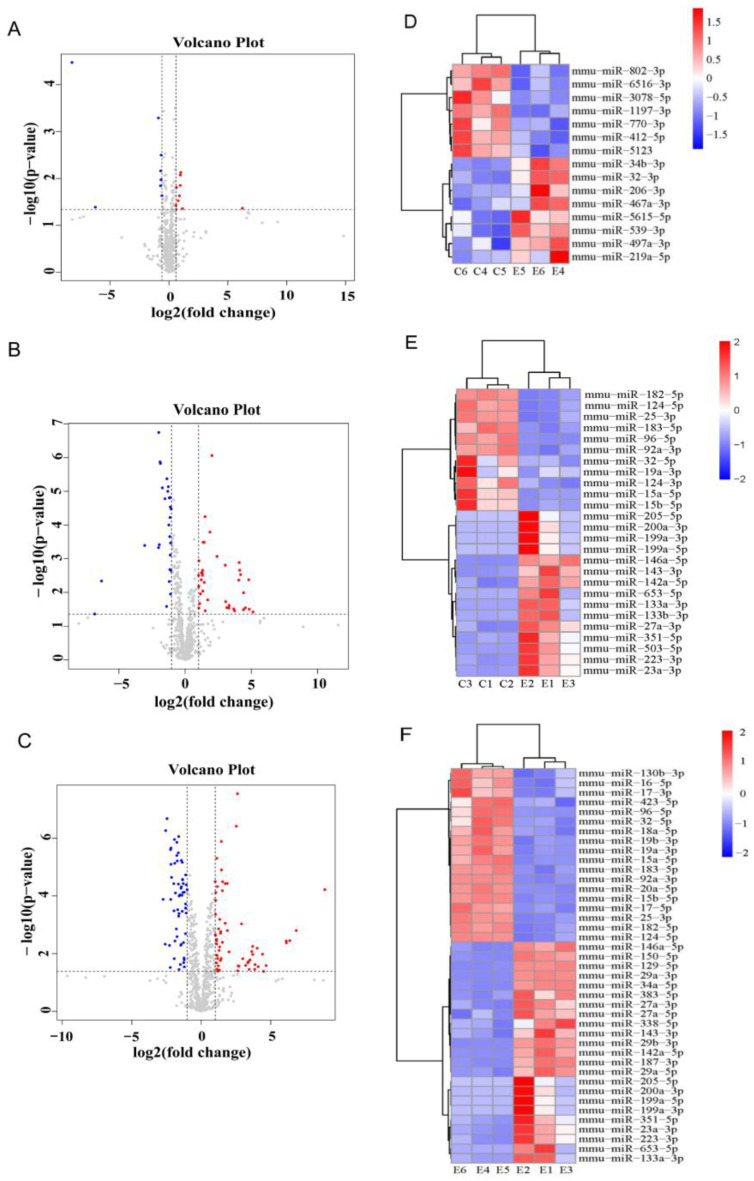
miRNA expression profiling in rd1 mouse retinas. (**A**,**B**) Volcano plots showing differentially expressed miRNAs in rd1 versus wild-type retinas at (**A**) P9 and (**B**) P14 (red: upregulated, log2FC ≥ 1.5; blue: downregulated, log2FC ≤ −1.5; gray: nonsignificant; adjusted *p* < 0.05). The plots are to show the differentially expressed miRNAs at the initiation and peak time of apoptosis in rd1 mice. (**C**) Temporal expression changes between rd1 retinas at P14 and P9 (log2FC ≥ 2). The plot is to track the progressive changes in key miRNA levels across multiple time points to delineate their dynamics during disease progression. (**D**–**F**) Hierarchical clustering of miRNA expression: (**D**) P9 rd1 vs. control, (**E**) P14 rd1 vs. control, and (**F**) P14 vs. P9 rd1. Heatmap colors represent Z-score normalized expression levels (red: high; blue: low). *n* = 3 biological replicates per group.

To identify miRNAs contributing to retinal degeneration in rd1 mice, miRNA sequencing data were analyzed to identify differentially expressed miRNAs in the retinas of rd1 mice at postnatal days 9 (P9) and 14 (P14). P9 and P14 were chosen because they are the initiation and peak time of apoptosis in rd1 mice. The volcano plots below illustrate the upregulated and downregulated miRNAs (Figure 4A–C). Differential miRNAs between rd1 and control mice at P9 and P14 were identified using a logFC threshold of ≥1.5 and a *p*-value < 0.05. Additionally, differential miRNAs between P14 and P9 rd1 retinas were screened using a logFC threshold of ≥2 and a *p*-value < 0.05.

At P9, we identified 10 upregulated and 9 downregulated miRNAs in rd1 mice compared to normal controls (Figure 4A). By P14, this increased to 40 upregulated and 27 downregulated miRNAs (Figure 4B, Table S3). Furthermore, 65 upregulated and 58 downregulated miRNAs were found in rd1 retinas at P14 compared to at P9 (Figure 4C).

At P14, miR-143-3p, miR-133a-3p, miR-133b-3p, miR-23a-3p, miR-142a-5p, miR-223-3p, and miR-653-5p were significantly upregulated, while miR-182-5p, miR-183-5p, miR-96-5p, miR-124-3p, and miR-25-3p were significantly downregulated. The heatmaps presented in Figure 4D–F visually represent these differentially expressed miRNAs. These findings suggest that these miRNAs may play a role in the retinal degeneration process that occurs in rd1 mice by regulating target genes involved in key biological pathways.

**Figure 5:**
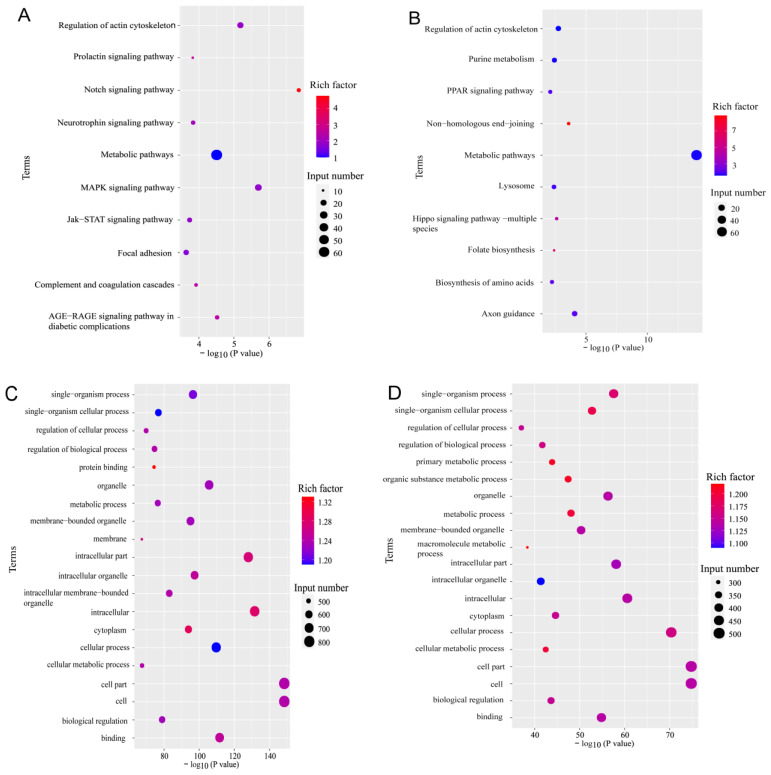
Bioinformatic analysis of miRNA target genes. (**A**,**B**) KEGG pathway enrichment analysis for targets of (**A**) upregulated and (**B**) downregulated miRNAs (top 10 pathways shown, ranked by −log10(*p*-value)). (**C**,**D**) Gene Ontology (GO) term enrichment for targets of (**C**) upregulated and (**D**) downregulated miRNAs, showing top biological processes. Dot size represents gene count; color intensity indicates rich factors.

The target genes of the differentially expressed miRNAs in rd1 mice at postnatal day 14 were significantly enriched in signaling pathways such as NOTCH, MAPK, PPAR, and HIPPO, which are closely associated with retinal diseases (Figure 5A,B). Additionally, we found that the target genes of these miRNAs were significantly enriched in biological regulation and cellular metabolism pathways (Figure 5C,D). These results suggest that the differentially expressed miRNAs may play a critical role in the progression of retinal degeneration in rd1 mice by modulating these pathways.

### 3.5. Differentially Expressed miRNAs in rd1 Mouse Retinas

**Figure 6:**
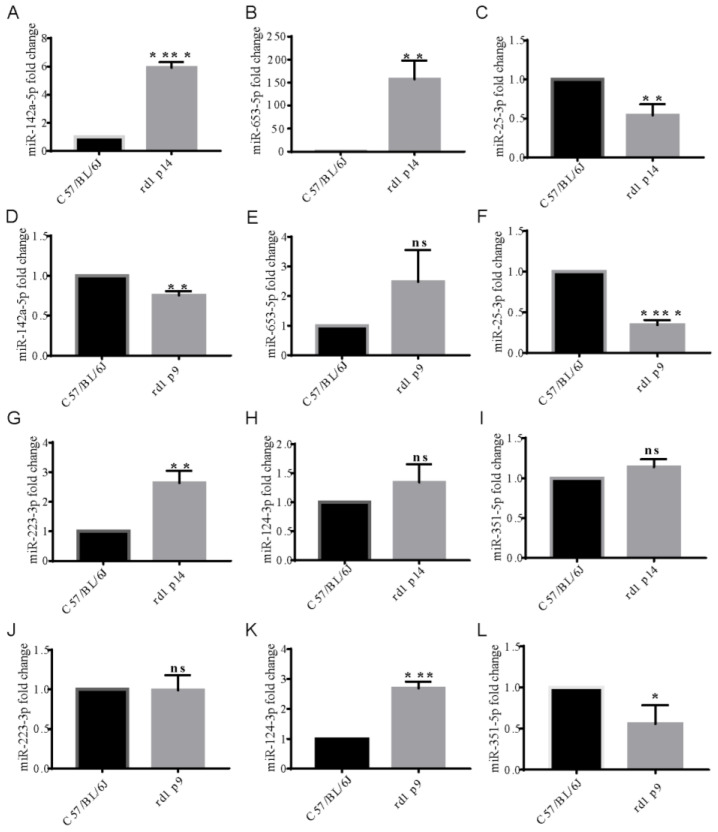
Validation of differentially expressed miRNAs by qRT-PCR. (**A**–**F**) Expression levels of miR-142a-5p, miR-653-5p, and miR-25-3p in the retinas of rd1 mice at P14 (**A**–**C**) and P9 (**D**–**F**); (**G**–**L**) expression levels of miR-223-3p, miR-124-3p, and miR-351-3p in the retinas of rd1 mice at P14 (**G**–**I**) and P9 (**J**–**L**). Data normalized to U6 snRNA and presented as mean ± SD fold change (*n* = 3 biological replicates; each replicate pooled from 2 retinas). * *p* < 0.05, ** *p* < 0.01, *** *p* < 0.001, **** *p* < 0.0001 by unpaired *t*-test versus age-matched controls; ns: not significant.

Six miRNAs—miR-142a-5p, miR-223-3p, miR-25-3p, miR-653-5p, miR-351-5p, and miR-124-3p—were identified as being involved in inflammatory responses, apoptosis, autophagy, and other processes closely associated with RP [19,20,21,22,23,24,25,26,27,28]. To validate these findings, we quantified the expression levels of these miRNAs using qPCR. The results revealed that miR-142a-5p expression in rd1 mouse retinas at postnatal day 14 was approximately 5.91-fold higher than in the control group (Figure 6A, *p* < 0.0001), while miR-223-3p expression increased by approximately 2.63-fold (Figure 6G, *p* < 0.01). In contrast, miR-25-3p expression decreased by approximately 0.54-fold (Figure 6C, *p* < 0.01), and miR-653-5p expression increased by approximately 157-fold (Figure 6B, *p* < 0.01). These expression trends were consistent with the miRNA sequencing results, suggesting that miR-142a-5p, miR-223-3p, miR-25-3p, and miR-653-5p may play significant roles in the retinal degeneration process that occur in rd1 mice. We also confirmed the change in miR-142a-5p expression by TaqMan method (Figure S3).

### 3.6. Analysis of Target Genes for Differentially Expressed miRNAs

**Figure 7:**
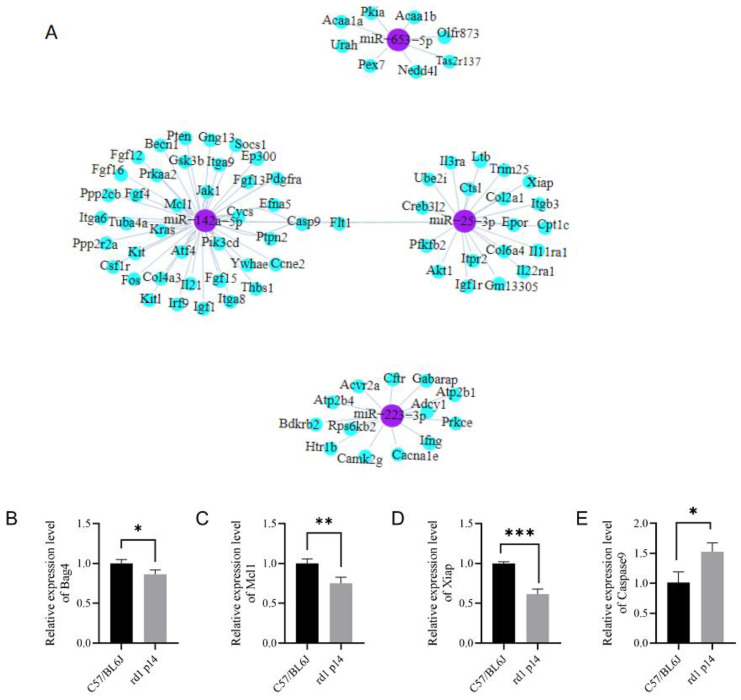
Bioinformatic prediction of miRNA–mRNA interaction networks and qPCR confirmation of predicted gene expression. (**A**) Computational analysis of target genes for validated miRNAs (miR-142a-5p, miR-223-3p, miR-25-3p, and miR-653-5p) using RNAhybrid and miRanda algorithms. Nodes represent miRNAs (purple circles) or their predicted target genes (blue circles), with edge thickness indicating interaction confidence score. Network visualized using Cytoscape 3.8.2 with force-directed layout. (**B**–**E**) qPCR analysis of the mRNA expression of genes, including Bag4 (**B**), Mcl1 (**C**), Xiap (**D**), Casp9 (**E**). (*n* = 3 biological replicates; each replicate pooled from 2 retinas). * *p* < 0.05, ** *p* < 0.01, *** *p* < 0.001 by unpaired *t*-test versus age-matched controls.

MicroRNAs exert their biological functions by binding to target mRNAs and downregulating their expression levels. To explore the mechanisms underlying the roles of miR-142a-5p, miR-223-3p, miR-25-3p, and miR-653-5p, we predicted their potential target genes using the RNAhybrid and miRanda databases (Figure 7A).

For miR-142a-5p, the predicted target genes included Kitl, Kit, Bag4, Flt1, Efna5, and Mcl1. Among these, Bag4 and Mcl1 are involved in anti-apoptotic responses [29,30], and the Kitl/Kit signaling pathway has been closely linked to retinal diseases [31]. For miR-25-3p, target genes such as Xiap and Casp9 are known to play critical roles in apoptosis [32,33]. Among the target genes of miR-223-3p, Bdkrb2 is associated with inflammatory responses [34], while Prkce functions as an anti-apoptotic gene [35]. Additionally, Nedd4l, a target of miR-653-5p, is closely related to autophagy [36]. Many of these target genes are involved in biological processes directly linked to RP [28], suggesting that differentially expressed miRNAs may contribute to the retinal degeneration process in rd1 mice by regulating these genes. We further confirmed the expression level of predicted miRNA targeted genes. qPCR analysis revealed that the mRNA expression levels of miR-142-5p target genes—Bag4, Mcl1, Xiap—decrease in retina from rd1 mice, suggesting that they are target genes of miR-142-5p. The expression level of Casp9 increases in rd1 mice, suggesting that Casp9 is regulated by other factors than miR-142a-5p (Figure 7B–E).

### 3.7. Knocking Down miR-142a-5p Slowed the Progression of Retinal Degeneration in rd1 Mice

**Figure 8:**
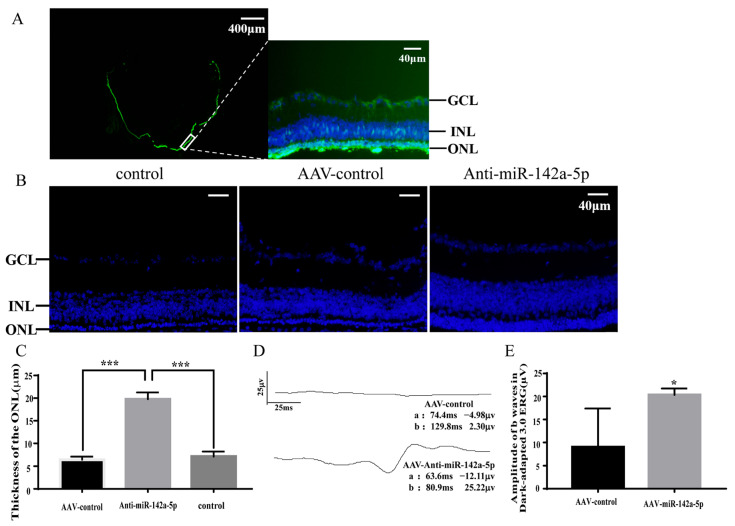
Therapeutic effects of miR-142a-5p knockdown in rd1 mice. (**A**) Retinal EGFP expression confirming viral transduction, the left panel shows a whole-mount view and the right panel shows a zoom-in view of white box showing the EGFP expression by scAAV. (**B**) Retinal morphology by DAPI staining showing preserved ONL structure. (**C**) Quantification of ONL thickness (μm) demonstrating significant rescue. (**D**,**E**) Functional improvement assessed by ERG: (**D**) representative b-wave traces and (**E**) amplitude quantification under dark-adapted 3.0 cd·s/m^2^ stimulation. Data represent mean ± SD (*n* = 3–4 mice/group; * *p* < 0.05, *** *p* < 0.001 by unpaired *t*-test). Experimental groups: untreated rd1 (Control), AAV-EGFP (AAV-control), and AAV-anti-miR-142a-5p (Anti-miR-142a-5p).

Since miR-142a-5p displayed a time dependent upregulation during RP progression and it has been suggested to regulate apoptosis, it was chosen for functional study to prove the pathological significance of the miRNAs identified in this study. On postnatal day 9, when rd1 retina starts to show apoptosis, rd1 mice were injected with either a knockdown miR-142a-5p scAAV viral vector or a control virus. The expression of enhanced green fluorescence protein (EGFP) in the retina was observed using fluorescence microscopy. EGFP was widely expressed throughout the retina, with the majority of its expression localized to the photoreceptor cell layer (Figure 8A).

Compared to untreated rd1 mice and those injected with the control virus, the thickness of the ONL was significantly increased in mice injected with miR-142a-5p knockdown (Figure 8B,C; *p* < 0.001). Additionally, retinal function was significantly improved in these mice compared to the control virus group (Figure 8D,E; *p* < 0.05). These results suggest that inhibiting miR-142a-5p expression in the retina can delay the degeneration of photoreceptor cells in rd1 mice.

## 4. Discussion

MicroRNAs are small non-coding RNA molecules, approximately 19–25 nucleotides in length, that regulate gene expression by binding to the 3′ untranslated region (3′-UTR) of target mRNAs, leading to their degradation or translational repression [37]. Numerous studies have demonstrated that the expression of many miRNAs is significantly altered in retinal diseases. For example, miR-155 expression is markedly increased in the retinas of mice with photooxidative damage, and treatment with miR-155 inhibitors has been shown to improve retinal function [38]. Similarly, miR-409-5p is upregulated in diabetic retinal tissues and high glucose-induced mouse retinal microvascular endothelial cells, and its knockdown reduces retinal neovascularization [39].

In this study, we observed significant changes in miRNA expression patterns in the retinas of rd1 mice through miRNA sequencing, identifying numerous miRNAs with potential therapeutic relevance for RP. These findings provide a foundation for exploring new targets for RP treatment. Among the differentially expressed miRNAs, we focused on miR-142a-5p, miR-223-3p, miR-25-3p, miR-653-5p, miR-351-5p, and miR-124-3p, which were further validated using qPCR. The results confirmed that miR-142a-5p, miR-223-3p, and miR-653-5p were significantly upregulated in the retinas of rd1 mice at postnatal day 14, while miR-25-3p was downregulated. These findings were consistent with the miRNA sequencing data.

miR-142a-5p, which is highly expressed in the retinas of rd1 mice, has also been found to be upregulated in four other RP models: P347S-Rhodopsin-transgenic mice, Δ307-rds mice, Rho−/− mice, and rds null mutant (rds−/−) mice [40]. This suggests that elevated miR-142a-5p expression may promote retinal degeneration in RP. To test this hypothesis, we knocked down miR-142a-5p in the retinas of rd1 mice using scAAV. We observed a significant thickening of the retinal photoreceptor layer and improved retinal function, confirming that increased miR-142a-5p expression exacerbates retinal damage in rd1 mice.

Anti-apoptotic genes such as Xiap, Birc3, Bcl2, Bcl2l2, and Mcl1 have been identified as target genes of miR-142a-5p [41]. In a canine RP model caused by mutations in the PDE6B gene, Xiap expression was downregulated [42]. Researchers have also demonstrated that the overexpression of Xiap in Line 1 P23H and Line 4 S334ter transgenic rats protects retinal photoreceptor cells [43]. These findings suggest that miR-142a-5p likely promotes photoreceptor apoptosis in RP by downregulating Xiap expression. Our study by qPCR analysis found that the expression level of Xiap decreases in the retina of rd1 mice, strongly suggesting that Xiap is a target gene of miR-142a-5p. Additionally, our miRNA sequencing data analysis predicted other target genes of miR-142a-5p, including Kitl, Kit, and Bag4. Following photodamage, photoreceptor cells activate the tyrosine kinase receptor (Kit) pathway by upregulating Kitl expression. This leads to the accumulation of the transcription factor Nrf2 and the induction of the antioxidant gene Hmox1, which helps counteract oxidative damage [31]. Furthermore, Bag4 has been reported to function as an anti-apoptotic gene [30]. Thus, miR-142a-5p may also contribute to RP progression by downregulating Kitl, Kit, and Bag4 expression, although further research is needed to confirm these mechanisms.

Studies have reported that miR-25-3p in mesenchymal stem cell-derived exosomes can target pro-apoptotic genes such as Phosphatase and tensin homolog (PTEN) and Fasl to protect cardiomyocytes [19]. Additionally, miR-25-3p has been shown to modulate pyroptosis in high glucose-induced retinal pigment epithelial cells through the PTEN/AKT signaling pathway [44]. In our analysis, we predicted that potential target genes of miR-25-3p include caspase9 (a pro-apoptotic gene) and AKT1 (serine/threonine-protein kinase 1), with AKT1 playing a role in the antioxidant processes of retinal ganglion cells [45]. These findings suggest that miR-25-3p may accelerate the degeneration of retinal photoreceptors in rd1 mice by regulating the expression of these genes.

Currently, miRNA mimics and inhibitors are being actively investigated as promising therapeutic agents to counteract miRNA dysregulation and ameliorate disease outcomes [46,47]. Our findings position miR-142a-5p inhibition as a potential therapeutic strategy for retinitis pigmentosa. We fully acknowledge that translating miRNA-based therapeutics into clinical practice faces several challenges, including efficient and targeted in vivo delivery, optimizing safety and specificity, and overcoming barriers in clinical translation. Nevertheless, the primary aim of our study was to identify a novel miRNA target for RP intervention. Should these broader challenges in miRNA drug development be overcome, miR-142a-5p inhibition holds compelling potential to evolve into a viable treatment for retinal degeneration.

## 5. Conclusions

In conclusion, our study provides a comprehensive profile of miRNA dysregulation in an rd1 mouse model of RP, identifying numerous miRNAs with altered expression during the phase of peak photoreceptor degeneration. We specifically pinpointed miR-142a-5p as a critically upregulated miRNA contributing to disease pathogenesis. We demonstrate that directly inhibiting miR-142a-5p protects against retinal cell death and preserves vision, establishing its crucial role in disease progression. These findings not only advance our understanding of RP’s molecular mechanisms but also directly suggest that miR-142a-5p is a promising therapeutic target for developing treatments to halt vision loss in this and potentially other inherited retinal diseases.

## Data Availability

The datasets supporting this article are available from the corresponding author on reasonable request. Our RNA-seq sequencing data has been uploaded to the GSA archive (CRA010104).

## Supplementary Materials

The following supporting information can be downloaded at: https://www.mdpi.com/article/10.3390/biology15020134/s1, Figure S1: Line chart of the retinal photoreceptor apoptotic index (%) of rd1 mice at different time points. Data represent mean ± SD (n = 3 mice/group); Figure S2: Representative TUNEL/DAPI-stained retinal sections at indicated postnatal days (40× magnification; scale bar = 40 μm) of WT mice. Blue: DAPI nuclear counterstain (all nuclei). Green: TUNEL-positive cells (apoptotic nuclei); Figure S3: Expression level of miR-142-5p at P14 (B) and P9 (A) of WT and rd1 mice. The miRNA level was quantified by TaqMan method. Data represent mean ± SD (n = 3 mice/group). * *p* < 0.05, ** *p* < 0.01 by unpaired *t*-test; Table S1: Primer sequences for miRNA; Table S2: Primer sequences for mRNA; Table S3: Upregulated and downregulated miRNA at P14 rd1 mice.

## Abbreviations

The following abbreviations are used in this manuscript:

RP	retinitis pigmentosa
RdCVF	rod-derived cone viability factor
miRNAs	microRNAs
DR	diabetic retinopathy
cGMP	cyclic guanosine monophosphate
ONL	outer nuclear layer
INL	inner nuclear layer
GCL	ganglion Cell Layer
ERG	electroretinography
ffERG	full-field electroretinogram
EGFP	enhanced green fluorescent protein
3′-UTR	3′ untranslated region
PTEN	phosphatase and tensin homolog
AKT1	serine/threonine-protein kinase 1
