# Supplementary material for: RNA Sequencing and Targeted Knockdown Reveal miR-142a-5p as a Driver of Retinal Degeneration in rd1 Mice
Source: Biology (Basel). 2026 Jan 13;15(2):134. doi: 10.3390/biology15020134 (PMC12837277; doi:10.3390/biology15020134)
Supplement: Supplementary file 1 [file biology-15-00134-s001.zip › biology-3884252-supplementary.pdf]

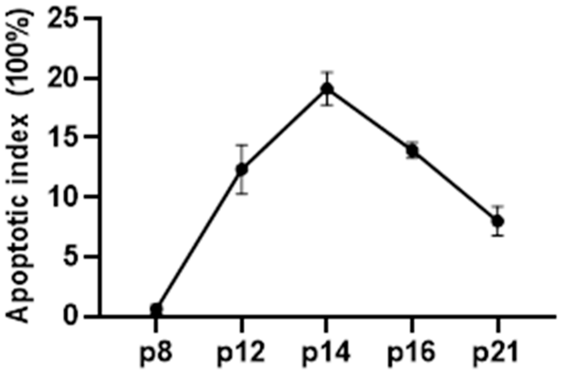

Supplementary Figure S1. Line chart of the retinal photoreceptor apoptotic index (%) of rd1 mice at different time points. Data represent mean ± SD (n=3 mice/group).

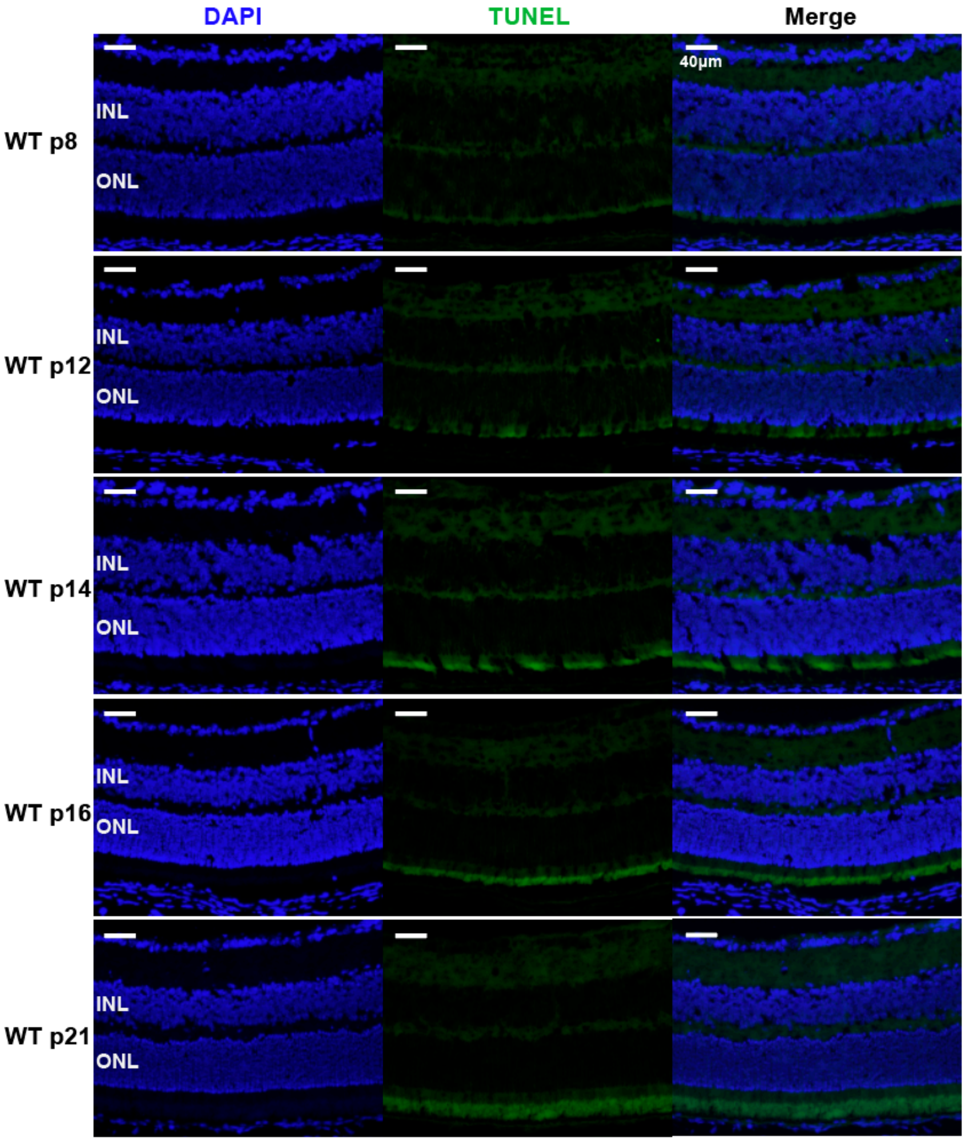

Supplementary Figure S2. Representative TUNEL/DAPI-stained retinal sections at indicated postnatal days (40× magnification; scale bar = 40 μm) of WT mice. Blue: DAPI nuclear counterstain (all nuclei). Green: TUNEL-positive cells (apoptotic nuclei).

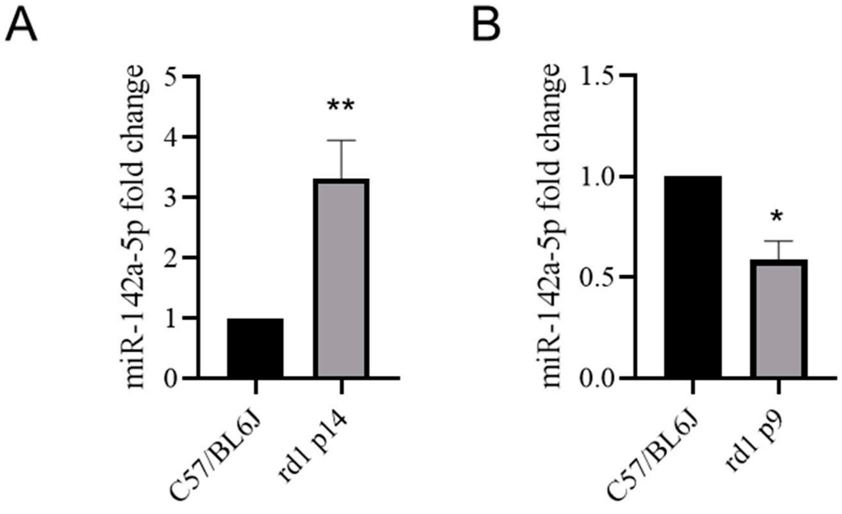

Supplementary Figure S3. Expression level of miR-142-5p at P14 (B) and P9 (A) of WT and rd1 mice. The miRNA level was quantified by TaqMan method. Data represent mean ± SD (n=3 mice/group). *P < 0.05, **P < 0.01 by unpaired t-test.

Table S1. Primer sequences for miRNA

MicroRNA	Primers for reverse transcription	Forward primers
microRNA-124-3p	GTCGTATCCAGTGCAGGGTCCGAGGTATTCGCACTGGATACGACGGCATT	GTAGAGGGTCTCGGGGATGT
microRNA-142a-5p	GTCGTATCCAGTGCAGGGTCCGAGGTATTCGCACTGGATACGACAGTAGT	GCGCGCATAAAGTAGAAAGC
microRNA-351-5p	GTCGTATCCAGTGCAGGGTCCGAGGTATTCGCACTGGATACGACCAGGCT	GTCCCTGAGGAGCCCTTTG
microRNA-223-3p	GTCGTATCCAGTGCAGGGTCCGAGGTATTCGCACTGGATACGACTGGGGT	GCGCGTGTCAGTTTGTCAAAT
microRNA-653-5p	GTCGTATCCAGTGCAGGGTCCGAGGTATTCGCACTGGATACGACCAGTAG	GCGCGGTGTTGAAACAATCT
microRNA-25-3p	GTCGTATCCAGTGCAGGGTCCGAGGTATTCGCACTGGATACGACTCAGAC	GCGCATTGCACTTGTCTCG

Table S2. Primer sequences for mRNA

Genes	Forward	Reverse
Bag4	AGCCCAACTCCAATGTCTCG	ATGGGGCAAGGTCATTCCAG
Mcl1	GGGGCAGGATTGTGACTCTT	GCATGTAGTTTGGTGGCTGG
Casp9	ACCTTCCCAGGTTGCCAATG	CCATTGCACTCCGGTCTTCT
Xiap	AGCCTCCTTAAACTTCGTGACA	TGGTGTCTGCAAGTACAAAAGTT C
Gapdh	GTGGCAAAGTGGAGATTGTTG	CGTTGAATTTGCCGTGAGTG

\

Table S3 Upregulated and downregulated miRNA at P14 rd1 mice

UP miRNAs	**Down miRNAs**
miR-1a-3p	miR-182-5p
miR-199a-3p	miR-183-5p
miR-143-3p	miR-96-5p
miR-152-3p	miR-124-3p
miR-199b-3p	miR-25-3p
miR-133a-3p	miR-124-5p
miR-142a-3p	miR-19a-3p
miR-27a-3p	miR-15a-5p
miR-146a-5p	miR-15b-5p
miR-23a-3p	miR-872-5p
miR-206-3p	miR-182-3p
miR-142a-5p	miR-92a-3p
miR-199a-5p	miR-449a-5p
miR-503-5p	miR-32-5p
miR-145a-5p	miR-183-3p
miR-199b-5p	miR-96-3p
miR-143-5p	miR-130b-5p
miR-299a-3p	novel_55
miR-455-5p	miR-20a-3p
miR-200a-3p	miR-92a-1-5p
miR-299a-5p	miR-670-3p
miR-223-3p	miR-1968-5p
miR-351-5p	miR-3084-3p
miR-141-3p	novel_27
miR-145a-3p	miR-9769-3p
miR-205-5p	novel_180
miR-455-3p	novel_200
miR-574-3p	
miR-133b-3p	
miR-133a-5p	
miR-486b-5p	
miR-486a-5p	
miR-429-3p	
miR-200b-3p	
miR-200c-3p	
miR-100-3p	
miR-10b-5p	
miR-1a-1-5p	
miR-27a-5p	
miR-653-5p	
